# Non-technical skills: a review of training and evaluation in urology

**DOI:** 10.1007/s00345-019-02920-6

**Published:** 2019-09-17

**Authors:** Cora Griffin, Abdullatif Aydın, Oliver Brunckhorst, Nicholas Raison, Muhammad Shamim Khan, Prokar Dasgupta, Kamran Ahmed

**Affiliations:** 1 MRC Centre for Transplantation, Guy’s Hospital, King’s College London, London, UK; 2 Department of Urology, Guy’s and St. Thomas’ NHS Foundation Trust, King’s Health Partners, London, UK; 3 Department of Urology, King’s College Hospital NHS Foundation Trust, King’s Health Partners, London, UK

**Keywords:** Non-technical skills, Training, Assessment, Debriefing, Urology

## Abstract

**Purpose:**

With non-technical skills (NTS) deficits being recognised as a major cause for error in surgery, there is an increasing interest in their training and evaluation. A growing number of training courses are emerging and some NTS curricula have also been created. Many different training methods are described in the literature but there is still uncertainty with regards to their optimum combination within a curriculum.

**Methods:**

A literature review of the electronic database Medline was performed. All articles published before December 2018 were screened by abstract and included if deemed relevant by the author. The included articles’ reference lists were also screened for further relevant studies.

**Results:**

Simulation training is accepted as the most effective way to train NTS. Within simulation training, it is shown that the ‘igloo’ full immersion/distributed simulation environment is appropriate for teaching NTS in urological scenarios where a designated operating room or space is not available. The use of multiple settings, for example wards and clinics as well as the operating room, is advantageous, as is training in an interprofessional team. Classroom teaching also plays a role in NTS training as an adjunct to simulation, with evidence that it improves some parameters of NTS. All levels, including qualified surgeons, benefit from NTS training; however, adaptation to both trainee level and specialty is important. Although less time consuming, training juniors and seniors together mainly benefits juniors, and training NTS at the same time as technical skills detracts from the quality of teaching. Debriefing is an important part of training and should be well structured; there are many debriefing models in existence, allowing for choice of method based on examiner preference and participant demographic. Furthermore, examiners should be well briefed in their task and trained in NTS assessment.

**Conclusion:**

To move forward, studies should combine tried and tested learning techniques into a curriculum covering all training levels, which should then be validated and followed up long term to ensure a positive impact on patient safety.

## Introduction

Non-technical skills (NTS) are the cognitive and social abilities that complement a clinicians’ technical ability and generally comprise decision making, leadership, team work, and situational awareness (Table 1) [1, 2]. They are often grouped into three categories: social skills (leadership, communication and teamwork), cognitive skills (decision making and situational awareness), and personal resource factors. The latter includes how individuals cope with stressors and fatigue, known to negatively impact NTS and technical performance (2].

**Table 1:** Non-technical skills (NTS)

Skill	Description
Decision making	The ability to diagnose, assess and implement decisions
Leadership	Appropriate and effective management of team members
Team work	Appropriate communication with ones’ team, remaining receptive to team members’ suggestions
Situational awareness	The perception of patient status, anticipation of required actions, and awareness of surrounding staff

There are many ways to categorise NTS. Some important skills commonly used in simulation assessment scales are described below

In the last decade, NTS have emerged as a vital area for improvement within healthcare: they are a major cause for error, causing 86% of adverse events in open surgery [3, 4]. A UK-based evaluation of skills survey found that only 41% of urological trainees felt that their NTS training was sufficient for their first day of practice compared to 78% of specialists, with an even lower 25% of trainees believing their current NTS training was sufficient [5].

Clearly, NTS training must improve further and there is a need for standardised and validated training curricula, especially in newer fields such as robotic surgery [1, 3, 5]. This review aims to collate the current evidence base on NTS training, assessment, curricula and debriefing methods to aid and recommend current practice and future developments in urological training.

## Methods

A literature review of the electronic database Medline was performed. All articles published before December 2018 were screened by abstract and included if deemed relevant by the author. The included articles’ reference lists were also screened for further relevant studies.

Search terms included: non-technical skills AND training AND urology AND English[lang]; non-technical skills OR NTS AND simulation AND English[lang]; Debrief AND Simulation AND English[lang]. This was to find both articles specifically related to current NTS training in urology as well as further articles from other disciplines from which urological training can learn and improve.

## Non-technical skills training

### Cognitive training

Cognitive training is an established training method in areas such as aviation, the military, and sports [6]. Studies have shown that mental- or motor imagery (MI) can lead to lower stress levels and better error detection rates with technical skills (TS) training [6–8]. MI is a low-cost approach which involves mental rehearsal of a task with the aid of a script. A number of studies have suggested that MI has a positive effect on NTS, but no concrete benefit has been observed in the literature [6, 9].

### Classroom/didactic teaching

Classroom teaching is useful for introducing NTS concepts, self-reflection and changing the attitudes of trainees [10]. The effect of a single didactic NTS seminar on a group of medical students was investigated with an examined scenario and compared to a control group. The NTS group significantly improved their teamwork and situational awareness, as well as “handling errors”. However, both groups saw a reduction in stress and better decision making. Medical endpoints and patient outcome did not differ significantly between groups [11]. Thus, although didactic teaching has a role in NTS training, exemplified by this study, difficult cognitive skills such as decision making and planning require further training using other methods [11, 12]. Other beneficial classroom-based techniques include observing famous incidents and video analysis of self-performance [12].

### Simulation

Simulation is the most effective way to train NTS [10, 13]. It is critical for teaching communication skills and shows superiority to didactic lectures for self-perceived improvement [14]. Ounounou et al. [13] divide simulation-based NTS training into three main categories: full immersion/distributed simulation (FIDS), high fidelity OR simulation (HFORS) and crisis resource management (CRM).

#### Full immersion/distributed simulation

FIDS consists of a 360° inflatable and mobile “igloo” shell filled with operative equipment to create a realistic environment [15]. Brewin et al. [16] investigated the combined teaching of TS (TURP) and NTS within this environment and demonstrated face, content and construct validity. The course received positive qualitative feedback and the NTS of the experienced urologists were significantly better than those of trainees, upon a NoTECHS scale (see Assessment methods), but did not demonstrate transfer of skills to OR. The results of this study were further supported by Brunckhorst et al. [17], where novices who were taught ureteroscopy and NTS within an FIDS environment outperformed those taught using didactic methods, with the former group gaining significantly higher NoTSS scores. The “igloo” is, therefore, a viable alternative for NTS courses where an OR is not available.

#### High fidelity simulation

HFORS uses a simulated OR for training to assess non-technical surgical skills. Abdelshehid et al. [18] conducted a prospective cohort study in which urology residents undertook a laparoscopic partial nephrectomy simulation-based team-training scenario, using validated simulator models. They found that the level of urology training did significantly affect non-technical performance using the NoTSS score, thus showing construct validity. Lee et al. [19] conducted a study similar but alongside anaesthetic trainees. 94% of participating trainees thought that the session was useful and should be included in training, demonstrating face validity. Although HFORS proves to be beneficial, long-term follow-up showing transfer validity is consistently lacking in the literature.

#### Crisis resource management and ward-based scenarios

Training must address the surgeon’s wider role, including outside the OR, in clinics and wards, since different skills are required for different settings [20]. Many studies exclusively use the OR setting for simulation but there are some examples of simulated ward rounds, such as the UK-based urology boot camp [3], an intense 1-week training curriculum and course for newly starting urology trainees. Simulated wards and clinics have also been employed as part of a larger programme in the urology-focussed course by Khan et al. [21], using the ‘SimMan’ model, and for general NTS training with multiprofessional groups. These courses were all well received [21, 22]. Future courses could stand to gain from incorporating these ideas into a mixed curriculum comprising both OR and ward scenarios.

CRM training, which addresses NTS in emergency scenarios, has been used successfully in aviation and acute care and is associated with a reduction in errors. Truta et al. [23] created a single-day CRM course with both didactic and simulation training for interprofessional EM teams. It resulted in significant NTS improvement two months following the course in all aspects of NTS for all levels of training. Yee et al. [24] also evaluated one similar CRM intervention and showed improvement at 1 and 2 months in all NTS categories. However, other studies do not show this retention [23]. CRM training is incorporated into a wider urology curriculum by Khan et al. [21], which shows feasibility and acceptability. However, this study focussed on technical performance and lacks quantitative data on NTS. Although CRM may be better suited to emergency teams, it should be considered for urological training pending further studies.

#### Team training

Team training rather than individual training has been found useful by trainees, particularly for communication skills [25]. Interprofessional team working needs to be addressed to improve patient safety, involving the range of professions that compose an OR team benefits simulations by improving communication and decreasing anxiety between groups [22, 26, 27]. This effect is most relevant to junior trainees, but seniors should also be aware of the issues faced by the less senior staff in their teams [22].

#### Training for robotic surgery

Robotic surgery is a multidisciplinary, complex environment and, thus, requires further development of NTS alongside TS training [1, 4]. New challenges include the new technology, the surgeon being physically displaced from their team, a change in the surgeons’ role and additional staff members to communicate with. The team must adapt their interactions and maintain excellent situational awareness and communication, for example staying aware of patient status and any equipment failure [1, 4, 12, 28]. Other NTS areas to consider in robotic training include cognitive skills: technically demanding robotic procedures require a high level of decision making and planning. The assistant surgeon also has a higher responsibility as they are at the patients’ side without the lead surgeon [1, 12, 28]. VR simulation can be used for robotic NTS training to further skills [1]. Individual and team reactions to system errors can be simulated, repeated and assessed. The latest generation trainers (dV-Trainer™ and Robotix mentor™) have an option to train the assistant alongside the surgeon, which allows for the development and assessment of teamwork in hazard scenarios and troubleshooting skills [4]. Considering current literature, it is recommended that NTS and team training be learnt in simulation training, with or without VR aid, that can replicate common and emergency scenarios in robotic surgery. This should support structured assessments within the robotic surgical curricula [1].

### Logistics

The structured integration of NTS training into the curriculum is important to improve delivery in medical education, ensuring competence and improved patient outcomes [20]. However, this faces many issues, for example the working hour restrictions, limited personnel and considerable costs [29]. HFORS is particularly costly and can be restricted to bigger centres with better resources [30, 31]. To combat this, innovations such as the FIDS “igloo” and a mobile education unit (MEU) have been created to transport simulation to new or more rural places [30] and standardise teaching using the same mobile setting. The ‘igloo’ has been shown to have face, content and construct validity and is recommended for NTS training. The MEU was also well received by clinicians [5, 10, 13, 30]. It is also disputed whether higher fidelity, costly models result in greatly improved learning outcomes: one study shows no significant difference between the groups who trained with a low fidelity model (costing €14) compared to a high-fidelity model (costing €2600) [25, 32]. Studies have also been run in the nursing profession and although higher fidelity models gave a benefit with TS training, the evidence for NTS is less robust and lacks any long-term significance [33].

In the Dutch urological practical skill curriculum, theoretically optimum methods for learning were not always logistically viable ([29]. For example, frequent short training sessions (e.g. 1 h per week) are known to be better than longer, less frequent sessions (e.g. one afternoon per month), but are more difficult to organise. Similarly, consolidation of skills cannot exclusively be achieved in short courses, but they are much easier to organise [29, 34]. Non-compliance and under prepared supervisors are also a problem, especially when they may not have clear instructions or emphasis on the importance of preparation [29]. On this basis, significant planning, well in advance, is necessary if a curriculum is to be successful.

### Retention of knowledge

Transfer validity and skill retention are particularly difficult to investigate since following up participants is logistically challenging, resulting in many studies lacking these important data. Studies which do follow up participants show NTS retention for at least 2 months in current training programmes, in which some claiming trainees are still benefitting at 6 months, but others note that there is no significant difference between the NoTSS scores of surgeons who have previously undertaken NTS training versus those who have not. These studies often employ a second simulation session to record improvement and do not look at real performance in the OR [23, 26, 35]. The longer-term effects of any courses are yet to be shown, but repeat training is necessary to maintain skills after they are learnt initially and make sure they translate to practice [35]. This poses two unanswered questions: how frequent should NTS training be and how should ‘refresher’ courses be structured [23, 26]?

### Further considerations

All training methods could benefit from using real urological case discussions (e.g. previous difficult cases from experience) to explore the decision processes and learn from mistakes and successes [20, 30]. Complex unexpected patient death scenarios have been successfully tested in high-fidelity simulation, and case studies have been employed in other studies such as the S-TEAMS course [20, 26]. NTS can also be consolidated in the workplace using informal methods and by debriefing thoroughly after critical incidents: it is shown that debriefing in the OR benefits NTS development. This could stand in place of or aid refresher courses to make sure the skills learnt continue to be employed in practice [12].

Furthermore, during career transitions doctors gain greater decision-making autonomy, making it a time of increased stress resulting in higher patient mortality, evidenced by the transitioning handover periods of doctors [13, 36]. Junior staff perform poorly when they have higher cognitive workloads; therefore, training of NTS at junior level is important to reduce errors in these times of high stress [13, 20].

## Evaluation and assessment

Most rating scales for NTS have been thoroughly validated for use in surgical simulation [10]. The most popular and comprehensively validated are NoTSS (Non-Technical Skills for Surgeons), NOTECHS (NOn-TECHnical Skills) and OTAS (Observational Teamwork Assessment for Surgery) [10, 37]. For specialties such as urology, more specialised assessment scales can be necessary: so far the NoTSUS (Non-technical Skills for Urological Surgeons) scale has been developed, as well as the ICARS for robotic surgery. These are both based on NoTSS and have been validated for use in training [1, 28].

Wood et al. [38] evaluated NTS training tools for both individual surgeons and teams and concluded that NoTSS was the best scale when evaluating an individual, but the NOTECHS was optimum for assessing teams. This highlights the need to remember the context of the simulation when deciding on an assessment tool [4, 38]. Tools to assess individual NTS components were also discussed in the review, such as the ‘RATE’ tool for situational awareness training; these tools are useful if there is a need to train only one aspect of NTS, possibly useful for specialties such as robotic surgery due to their specific challenges [38].

To aid assessment, a ‘talk-aloud’ protocol can be adopted where participants narrate their considerations and decisions throughout the simulation [39]. Assessors are also able to take NoTSS as an independent course for training in scoring participants [35]. Both these elements can make assessment fairer and help standardise it.

## Debriefing

It is widely accepted that the main opportunity for long-lasting and deep learning of NTS takes place during debriefing after simulation [40]. A structured debriefing session with a skilled facilitator is thought to be vital for the acquisition of NTS, as it encourages self-reflection [10, 35]. Debriefing after real events is also beneficial but should be structured differently [41]. The specific elements of debriefing required for improvement in NTS are not clearly defined in healthcare, and the specific skills needed for effective debriefing after simulation for NTS training can be unclear when both TS and NTS training are reviewed together. Most current studies on debriefing are limited in application due to bias and lack of generalisability; however, advice on a more systematic and standardised approach to the debrief session is needed to ensure all participants in NTS courses benefit consistently from training [40, 42].

There are three distinct debriefing categories (Table 2): facilitator-guided post-event debriefing, self-guided post-event debriefing, and facilitator-guided within-event debriefing, e.g. freeze frames [43]. Generally, debriefing should be as long as the simulation itself, and there should be a briefing to let participants know what to expect [40]. Frameworks for debriefing conversations can be found summarised in the review by Sawyer et al. [43] but more research is needed to determine any concrete benefit from using one model over another: it is likely that any model can be effective, with the act of debriefing being the important part. It is also necessary to account for the simulations’ context and what the debriefer feels most confident with when deciding on the model [43].

**Table 2:** Debriefing styles

Debriefing technique	Comments
Instructor-led debriefing	Current ‘gold standard’
	Resource intensive (requires skilled facilitator)
	Can include other techniques
Self-debriefing	More cost effective than instructor-led debriefing
	Benefits more experienced trainees
Video recording review	Adjunct to other methods
	Lack of evidence supporting efficacy
Eye tracking technology	Improves patient safety tasks where visual cues must be recognised, e.g. checking patient’s wristband
Re-do stations	Face validity—found useful by students
	More evidence required
Freeze frames	Promote deliberate practice
	Adjunct to post-event debriefing only
Team debriefing	Potentially beneficial, more research needed to compare to individual debriefing

There are many different debriefing styles which can be employed for simulated scenarios. It is important to choose one which is both well suited to the type of scenario and that the examiner or instructor is comfortable using

Having a script or aid can help the debriefer to deliver a higher quality session by improving team leader performance; in a study by Jaye et al., a standardised structure (‘The Diamond’) was put forward to refocus debriefing on NTS rather than TS, as well as make sure the experiences between participants are equal [40, 44]. ‘The Diamond’ is a two-sided prompt sheet: the first side contains the scaffolding, with a series of specifically constructed questions for each phase of the debrief; the second lays out the theory behind the questions and the process. With this the variation in what is expected from debriefing can be addressed and standardised [44]. Assessment scales like NoTSS are also useful aids, either as an adjunct to discussion or self-reflection, giving the participant objective feedback to reflect on [38].

### Debriefing styles and techniques

It is thought that having a skilled debriefer is important to the concept, but recent studies show that this might not be the case, provided that there is a form of educational process post-simulation [40]. Self-debriefing (e.g. reviewing a video of oneself with an assessment scale like NoTSS) has been shown to be effective in learning CRM, and it was found to be similarly effective to traditional instructor debriefing in other simulations [31]. This is important, as it can be expensive to train and hire senior individuals to these roles which impact the feasibility of simulation sessions and curricula [40]. Self-assessment has also been shown to create more goals for learning, which are then more likely to be carried out by the individual [45]. Self-debriefing is more cost effective than instructor debriefing, however, with a higher budget, instructor debriefing is preferred [31]. For more experienced learners, there is less reliance on an instructor and self-assessment is more cost effective, as well as allowing participants to control the pace of debriefing and the opportunity for review of self-perceived weaknesses [43, 46].

Often video recording is used in debriefing; however, recent studies have failed to find any additional benefit from using this technique [40, 47]. It may even distract participants from focusing on the learning objectives, but current research is not robust enough to discount it [46]. Eye tracking technology is an innovative method to provide participants with feedback. It significantly improves certain practices compared to verbal feedback, but other behaviours, such as decision making, are unaffected [48]. Alternatives to traditional instructor debriefing include use of self-video review, multimedia debriefing, and within-team debriefing [31]. Re-do stations have also been found useful by students as learning and debriefing experience, and those who had a staff member present found it more useful [49].

Freeze frames are a form of facilitator-guided within-event debriefing [13]. This is described as ‘stop action’ debriefing—participants can be stopped when an error occurs and receive corrective feedback before trying again. This promotes deliberate practice. However, within-event feedback alone has been found to be inferior to post-event feedback; thus, post-event debriefing should always accompany this method [43].

A final consideration is whether debriefing should be just for the individual or within their team. In a study by Martin et al. [30], half of each group were active participants in a case while the other half observed, and both participants and observers were invited to take part in the debriefing sessions. Participants found this to be a safe and positive educational experience [30]. In another study, CRM scenarios were followed by an instructor facilitated debriefing, with both teams involved in each other’s debriefing [23]. Both group and individual methods are able to adequately facilitate learning; thus, the method used should be decided based on logistical considerations and preference of the examiner.

## Recommendations

To introduce NTS concepts, teaching should be classroom based; once this is completed and there is proficiency in basic technical skills, an appropriate form of simulation can be undertaken [12, 13]. There is evidence of this concept in the UK ‘core trainee boot camp’, where the idea of NTS and NoTSS assessment scale was introduced to early surgical trainees in the classroom, and in courses like ‘S-TEAMS’, where immediate post-graduate doctors were excluded since they would not have proficiency in technical skills [26, 36]. To date, the literature surrounding all forms of training has focused on surgical trainees and medical students rather than qualified surgeons, but new specialists can also become exhausted and burnt out due to poor NTS training and, in one Australian study, a decline in NTS was found post-fellowship for senior experienced surgeons [13, 35]. Considering that NTS do not always correlate with experience, there is evidently room for NTS training to be incorporated at higher stages of medical training [13, 25, 35]. It is useful to bear in mind that whilst training juniors and seniors together may be less time consuming and benefit juniors, it has a detrimental effect on the quality of teaching for seniors [29].

To create an NTS curriculum in urology, training should be specific to the level of seniority and subspecialty whilst remaining logistically viable [50]. To date, research has tended to focus on the validity and feasibility of specific aspects of NTS training, with high-quality studies evaluating transfer of NTS still lacking [35], which should be addressed in future studies. Simulation and CRM training improve all NTS categories at all levels; however, more research is also needed to find the relative benefit of different simulation settings (ward vs OR) for different subspecialties. Didactic teaching improves teamwork and situational awareness and should be used as an adjunct to simulation. Training bodies should investigate the NTS component deficits in the target audience (e.g. situational awareness in robotics) and adapt teaching accordingly (Table 3). Debriefing should not be overlooked: a combination of methods described may be used depending on the scenario and resources available. Any future training should thoroughly document and test their debriefing methods to improve the volume of literature on the topic. NTS teaching must be repeated and adapted throughout surgical training, with an effort to monitor and teach skills in the real OR: there should be research into creating resources given on training courses to achieve this.

**Table 3:** Summary of training modalities

Training modality	NTS components
Simulation and crisis resource management	All
Simulation: team training	Communication
Simulation: virtual reality (robotics)	Teamwork, cognitive skills
Simulation: case scenario	Decision making
Didactic	Teamwork, situational awareness
Didactic: case study	Decision making
Cognitive task analysis (CTA)	Decision making
Real OR debriefing	All

Different training modalities work best for training different aspects of non*-*technical skills; however, there is still room for further research into the relative benefit of different simulation settings (e.g. ward vs OR) on individuals

## Conclusion

NTS training has come a long way in the past decade, with many more courses offered for trainees and better validated training tools. A standardised surgical NTS curriculum is still lacking, facing logistical challenges alongside the issues of determining optimum training methods and testing validity. Simulation training is the most effective way to train NTS, with FIDS and lower fidelity models an acceptable alternative to HFORS. Didactic teaching improves some components of NTS and is a good adjunct to simulation, with a classroom-based element giving space for participant briefing and case-based discussion. Training TS alongside NTS removes the focus from the NTS aspect and is detrimental to learning; however, this may be avoided with adequate briefing for both participants and examiners. Debriefing is one of the most important elements of training, allowing for longer-term learning. There are many ways to debrief; the optimum debriefing method for any given training session is one which is well planned and adapted to the specific situation, bearing in mind the experience of the trainees and the trainers. Assessment scales give participants objective feedback on their performance and are useful when used by NTS trained examiners.

NTS training is appropriate for all levels, though training juniors alongside seniors mainly benefits the former; adaptation to level of seniority and specialty is beneficial. Future research will likely focus on the amalgamation and implementation of the well-described training methods into extensive, validated curricula aimed at all stages of a medical career.

## Abbreviations

CRM: Crisis resource management
CTA: Cognitive task analysis
DUPS: Dutch urological practical skills (curriculum)
MI: Mental/motor imagery
NoTSS: Non-technical skills for surgeons (assessment scale)
NTS: Non-technical skills
OR: Operating room
TS: Technical skills
VR: Virtual reality
